# ScGOclust: leveraging gene ontology to find functionally analogous cell types between distant species

**DOI:** 10.1093/bioinformatics/btaf195

**Published:** 2025-07-15

**Authors:** Yuyao Song, Yanhui Hu, Julian Dow, Norbert Perrimon, Irene Papatheodorou

**Affiliations:** European Bioinformatics Institute, European Molecular Biology Laboratory, Wellcome Genome Campus, Hinxton, CB10 1SD, United Kingdom; Department of Genetics, Harvard Medical School, Boston, MA 02115, United States; School of Molecular Biosciences, University of Glasgow, Glasgow G12 8QQ, United Kingdom; Department of Genetics, Harvard Medical School, Boston, MA 02115, United States; Howard Hughes Medical Institute, Chevy Chase, MD 20815, United States; European Bioinformatics Institute, European Molecular Biology Laboratory, Wellcome Genome Campus, Hinxton, CB10 1SD, United Kingdom; Earlham Institute, Norwich Research Park, Norwich, NR4 7UZ, United Kingdom; Norwich Medical School, University of East Anglia, Norwich NR4 7UA, United Kingdom

## Abstract

**Motivation:**

Basic biological processes are shared across animal species, yet their cellular mechanisms are profoundly diverse. Comparing cell-type gene expression between species reveals conserved and divergent cellular functions. However, as phylogenetic distance increases, gene-based comparisons become less informative. The gene ontology (GO) knowledgebase offers a solution by serving as the most comprehensive resource of gene functions across a vast diversity of species, providing a bridge for distant species comparisons.

**Results:**

Here, we present scGOclust, a computational tool that constructs *de novo* cellular functional profiles using GO terms, facilitating systematic and robust comparisons within and across species. We applied scGOclust to analyse and compare the heart, gut, and kidney between mouse and fly, and whole-body data from *Caenorhabditis elegans* and *Hydra vulgaris*. We show that scGOclust effectively recapitulates the function spectrum of different cell types, characterizes functional similarities between homologous cell types, and reveals functional convergence between unrelated cell types. Additionally, we identified subpopulations within the fly crop that show circadian rhythm-regulated secretory properties and hypothesize an analogy between fly principal cells from different segments and distinct mouse kidney tubules. We envision scGOclust as an effective tool for uncovering functionally analogous cell types or organs across distant species, offering fresh perspectives on evolutionary and functional biology.

**Availability and implementation:**

ScGOclust is publicly available on CRAN: https://cran.r-project.org/web/packages/scGOclust/index.html and development versions are available on GitHub: github.com/Papatheodorou-Group/scGOclust/.

## 1 Introduction

Vertebrates and invertebrates have functionally analogous organs, yet they exhibit substantial diversity in morphology and mechanisms. Single-cell atlases from various species (“Evolution at the cellular level” 2023) provide comprehensive gene expression profiles, enabling molecular comparisons of cell types and organ functions. While comparative studies among closely related species have progressed rapidly, analysing cell-type gene expression between evolutionarily distant species, such as those from different phyla, remains challenging due to the complexity of gene evolution. Gene homology mapping is often imprecise due to ambiguity, added on to the fact that analysis commonly excludes paralogous and species-specific genes (Song *et al.* 2023).

Recently, a few methods have been developed to improve the integration of single-cell RNA sequencing (scRNA-seq) data across distant species by refining gene homology mapping (Tarashansky *et al.* 2021, Rosen *et al.* 2024). Here, however, we take a different approach: rather than focusing on gene-level homology, we aim to identify higher-order features constructed from genes that are inherently shared across species. This perspective leads us to the gene ontology (GO) knowledgebase, the world’s largest repository of information on gene functions (Ashburner *et al.* 2000, Gene Ontology Consortium *et al.* 2023). GO terms cover three domains: molecular function (MF, molecular-level activities performed by gene products), cellular component (CC, the cellular location where a molecular function takes place), and biological process (BP, biological programs accomplished by the concerted action of multiple molecular activities), among which GO BP is the most relevant for cell type functional comparison. Importantly, GO terms are species-neutral and have consistent annotation standards between species (Rhee *et al.* 2008). Many GO annotations are manually curated and supported by experimental evidence, with orthology occasionally serving as a supplementary source. Therefore, we hypothesize that GO BPs can serve as a powerful bridge to study cell type functional similarities, particularly between phylogenetically distant species.

A few studies have employed a top-down approach to perform GO enrichment analysis on co-expressed orthologs between closely related species (Liu *et al.* 2021, Jung *et al.* 2023, Wang *et al.* 2023). Encouragingly, a study comparing Nematostella with vertebrates highlights their potential for cross-phyla cell type comparison (Sebé-Pedrós *et al.* 2018). Nonetheless, these analyses treat GO as a tool for *post hoc* validation rather than for discovery. Currently, no bioinformatics software exists that leverages cellular GO BP profiles to perform *de novo* identification of functionally similar cell types using scRNA-seq data, while correctly utilizing the information in and handling the structure of the GO (Rhee *et al.* 2008, Gaudet and Dessimoz 2017, Wijesooriya *et al.* 2022).

Here, we introduce scGOclust, a bioinformatics software to comprehensively detect cell types with similar functional profiles across remote species, using scRNA-seq data based on GO BP features. We implemented careful mechanisms to handle the features of the GO resource, such as their hierarchy and evidence codes, while leveraging single-cell data to characterize cell type functional profiles. We highlight the utility of scGOclust, emphasizing its distinctive ability to analyse evolutionarily distant species through a comparative study of the heart, gut, and kidney between mouse and fly, and whole-body atlas between *Caenorhabditis elegans* and *Hydra vulgaris*. We showcase the functional correspondence between homologous cell types and similarities between non-homologous cell types due to functional convergence.

## 2 Materials and methods

### 2.1 Datasets and preprocessing

We used the 10X, stringent datasets of the fly heart and gut from the Fly Cell Atlas (Li *et al.* 2022), which was downloaded from https://flycellatlas.org/. The fly renal system data (Xu *et al.* 2022) were downloaded from the Gene Expression Omnibus (GEO) database with Accession No. GSE202575. We downloaded raw count matrices for the mouse heart (McLellan *et al.* 2020) from the EBI Single Cell Expression Atlas (Moreno *et al.* 2022) No. E-MTAB-8810 (only the no compound treatment mouse data were used). The mouse stomach (Adult_Stomach) and intestine (Adult_intestine) data were downloaded from the Mouse Cell Atlas 2.0 (Han *et al.* 2018) and combined into the ‘mouse gut’ dataset. Immune cells in the mouse gut datasets were removed because there were no blood cell types in the fly data. The mouse kidney data were from GEO (Ransick *et al.* 2019) with Accession No. GSE129798. Fly stem cells were removed because there were no stem cells in the mouse data, in line with the original publication’s cross-species analysis. *Caenorhabditis elegans* whole-body scRNA-seq data (Ghaddar *et al.* 2023) were downloaded from Zenodo No. 7958249 and *H. vulgaris* adult dataset (Siebert *et al.* 2019) from Broad Single-Cell Portal No. SCP260. Mouse orthologs of fly genes are obtained from FlyBase (release FB2023_06) (Gramates *et al.* 2022) for the kidney analysis.

### 2.2 GO BP annotations of genes

The GO annotations of genes in mouse and fly were obtained from ENSEMBL (version 108) (Cunningham *et al.* 2022) using the R package biomaRt (V2.46.3) (Smedley *et al.* 2009). To infer sequence orthology-based GO annotations, eggNOG-mapper v2 (Cantalapiedra *et al.* 2021) was run for *C. elegans* genome WS260 downloaded from Wormbase and *H. vulgaris* genome Hydra2.0 downloaded from NHGRI. These two specific genome versions were used in scRNA-seq data reads mapping in the original publications. We did not find curated GO annotations in ENSEMBL or NCBI of these particular versions, so we used eggNOG-mapper v2 to demonstrate the flexibility to apply scGOclust on less-curated genomes.

### 2.3 Constructing cell type profiles of BP and cross-species comparison

**Figure 1.**
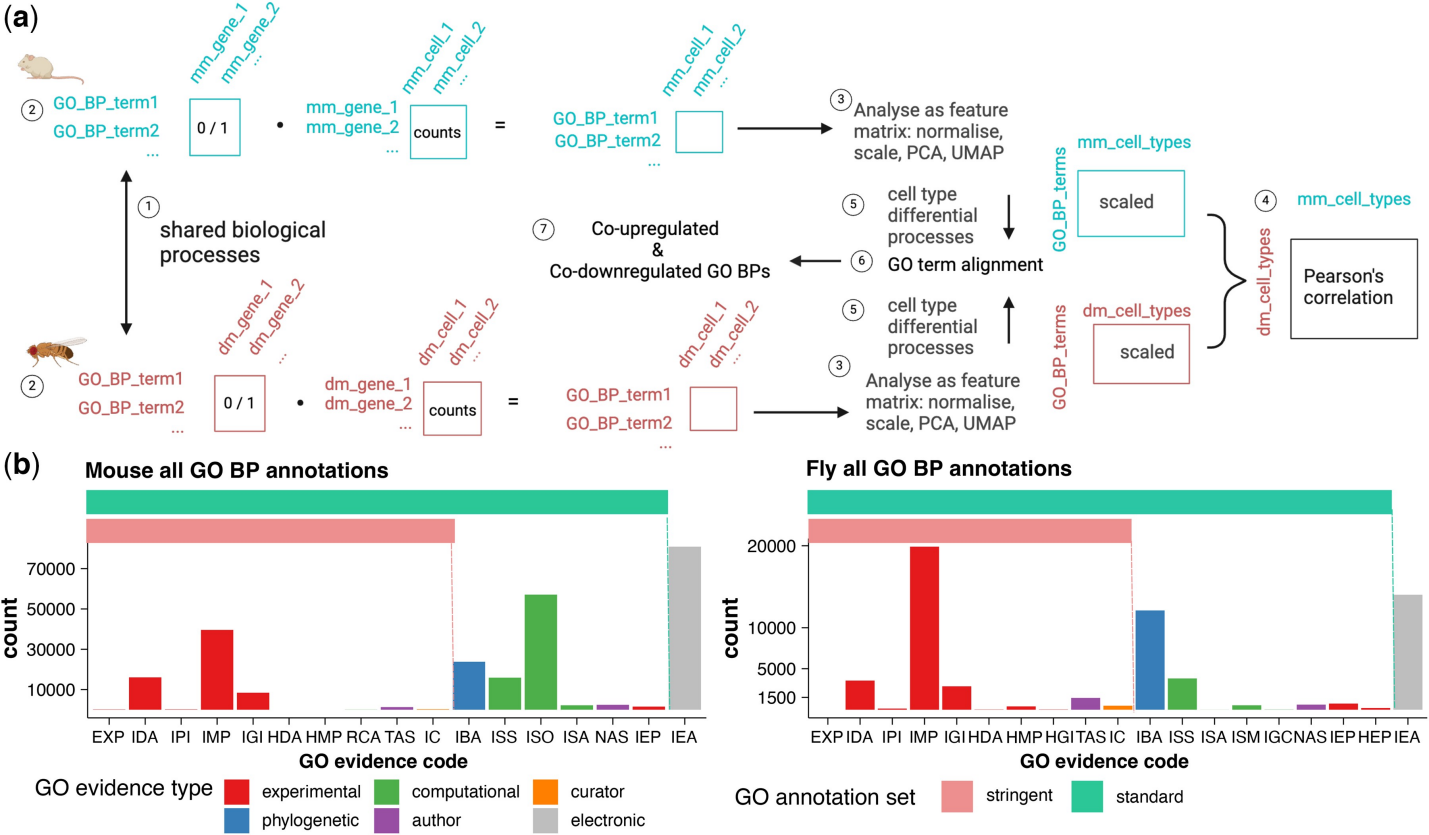
Schematic of the scGOclust method and overview of GO BP annotation sources in mouse and fly. (a) Schematic of scGOclust with details available in Section 2. (b) GO BP annotations are categorized by evidence code, highlighting the groupings used in scGOclust. GO: gene ontology; BP: biological process; PCA: principal component analysis; UMAP: Uniform Manifold Approximation and Projection; EXP: inferred from experiment; IDA: direct assay; IPI: physical interaction; IMP: mutant phenotype; IGI: genetic interaction; HDA: high-throughput direct assay; HMP: high-throughput mutant phenotype; HGI: high-throughput genetic interaction; RCA: reviewed computational analysis; TAS: traceable author statement; IC: curator inference; IBA: biological aspect of ancestor; ISS: sequence/structural similarity; ISO: sequence orthology; ISA: sequence alignment; ISM: sequence model; NAS: non-traceable author statement; IEP: expression pattern; HEP: high-throughput expression pattern; IEA: electronic annotation. Icons are from BioRender.com.

To construct a cellular functional profile based on gene expression and GO BP terms, scGOclust performs multiplication of (1) a gene expression count matrix of cells and (2) a binary matrix with GO BP annotations of genes (Fig. 1a). At this stage, we use the complete hierarchy of direct GO BP annotations from ENSEMBL by default to ensure that all information is preserved. This GO BP feature matrix is treated similarly to a count matrix in classic scRNA-seq analysis and is subjected to normalization, *z*-score scaling, and dimensionality reduction. Upon clustering of this profile, one can identify groups of cells with similar GO BP profiles. The method further performs differential GO BP analysis to identify the relative up- and down-regulation of BP terms in different cell types. To assess cell type similarity across species, the method starts with re-scaling the GO BP profiles from different species using only GO BP terms found annotated in both species, then calculating the average GO BP activity in each cell type. Subsequently, Pearson’s correlation coefficient is used to quantify the similarity of GO BP activity between cell type pairs. This approach highlights cell type pairs that exhibit similar activation and repression patterns of BPs. For the cell type pairs of interest, the method then pinpoints the exact terms that drive the observed similarity by calculating and harmonizing shared up-regulated and down-regulated terms.

### 2.4 Handling GO hierarchy

To handle the hierarchical structure of the GO, we adopted three approaches. Firstly, we calculate cell type GO BP profile correlations using normalized, *z*-score-scaled profiles. This scaling reduces the impact of broad, high-level terms that cover many genes and show uniformly high activity across cell types, as such terms yield small, invariant values in the scaled data. Secondly, to query enriched GO terms of a specific cell type, we refine the results to retain only the most specific terms by removing ancestors if their descendants are present. This is achieved using the ‘minimal_set’ function from the R package ontologyIndex (Greene *et al.* 2017). Finally, when identifying shared up-regulated or down-regulated terms between two cell types from two species, we use scOntoMatch (Song and Papatheodorou 2023) to determine the last common ancestor of the two lists of GO terms. This approach ensures that cases where one species has a more granular term and the other has its parent term are preserved. By doing so, we keep the complete and most specific BPs that show shared up- or down-regulation by the two cell types from different species. It is worth noting that we did not choose to limit the depth of GO BP terms while building the GO BP profile matrix because the information content of GO terms is not uniform—terms at the same depth can differ significantly in their granularity across BPs (Rhee *et al.* 2008). Theoretically, this may result in certain aspects of GO BP having lower granularity than others. However, this did not affect the analyses conducted in this study, and addressing the unevenness of GO BP is beyond the scope of this work.

### 2.5 GO evidence codes

We paid specific attention to allow for the selection of GO annotations based on evidence codes in scGOclust. The evidence codes (GO Consortium 2023) indicate how the annotations are supported and fall into six categories: experimental, phylogenetically-inferred, computational, author statement, curator statement, and electronic. The same record can be supported by several evidence codes. Figure 1b shows the total number of annotations grouped by evidence codes for mouse and fly as of the writing of this manuscript. After reviewing the evidence codes, we further selected some codes to form a standard and stringent set that we advise to use in scGOclust analysis, as shown in Fig. 1b. In the standard set, we removed electronic annotations since they are not manually reviewed, although the method is usually subjected to various quality assessments (GO Consortium 2023). We further provide the possibility to use the stringent set, in which phylogenetic and computational evidence codes, except RCA (reviewed computational analysis), were removed as they are not associated directly with experimental evidence. Non-traceable author statement code was also removed due to no original citations. We also removed those inferred from (high-throughput) expression patterns (IEP/HEP) because they were annotated solely based on expression change which makes it difficult to decide on whether they are directly linked to the process or an downstream event, though in practice, curators usually use these codes with caution. It is worth noting that while the stringent set might be interesting for some use cases, experimental code annotations do not necessarily imply a higher quality of the annotation and can be biased towards experimentally focused genes and can be essential for non-model species (Carbon *et al.* 2009, Gene Ontology Consortium *et al.* 2023).

### 2.6 Analysis and visualization of scRNA-seq data

We used the Seurat (V4.1.1) (Hao *et al.* 2021) framework to perform a standard analysis of the raw count matrix of scRNA-seq data for the mouse and fly gut and kidney with default parameters available in the scGOclust source code. Cell type differential expression analysis was performed with Wilcoxon signed-rank test with Bonferroni correction with FindAllMarkers. Adjusted *P*-value ≤.05 was considered statistically significant.

### 2.7 Comparing with SAMap on mouse and fly kidney data

We ran SAMap (v1.0.2) (Tarashansky *et al.* 2021) following the GitHub tutorial. Mouse genome version GRCm38 and fly genome r6.31 were used for BLAST as in the published data.

### 2.8 Comparing with a conventional *post hoc* analysis

We used the enrichGO function from ClusterProfiler (v3.18.1) (Wu *et al.* 2021) to perform GO BP enrichment using all cell type marker genes for each cell type in both species. GO terms with Bonferroni correction adjusted *P*-value ≤.01 were considered statistically significant.

## 3 Results

### 3.1 Functional similarities between fly and mouse heart cell types

**Figure 2.**
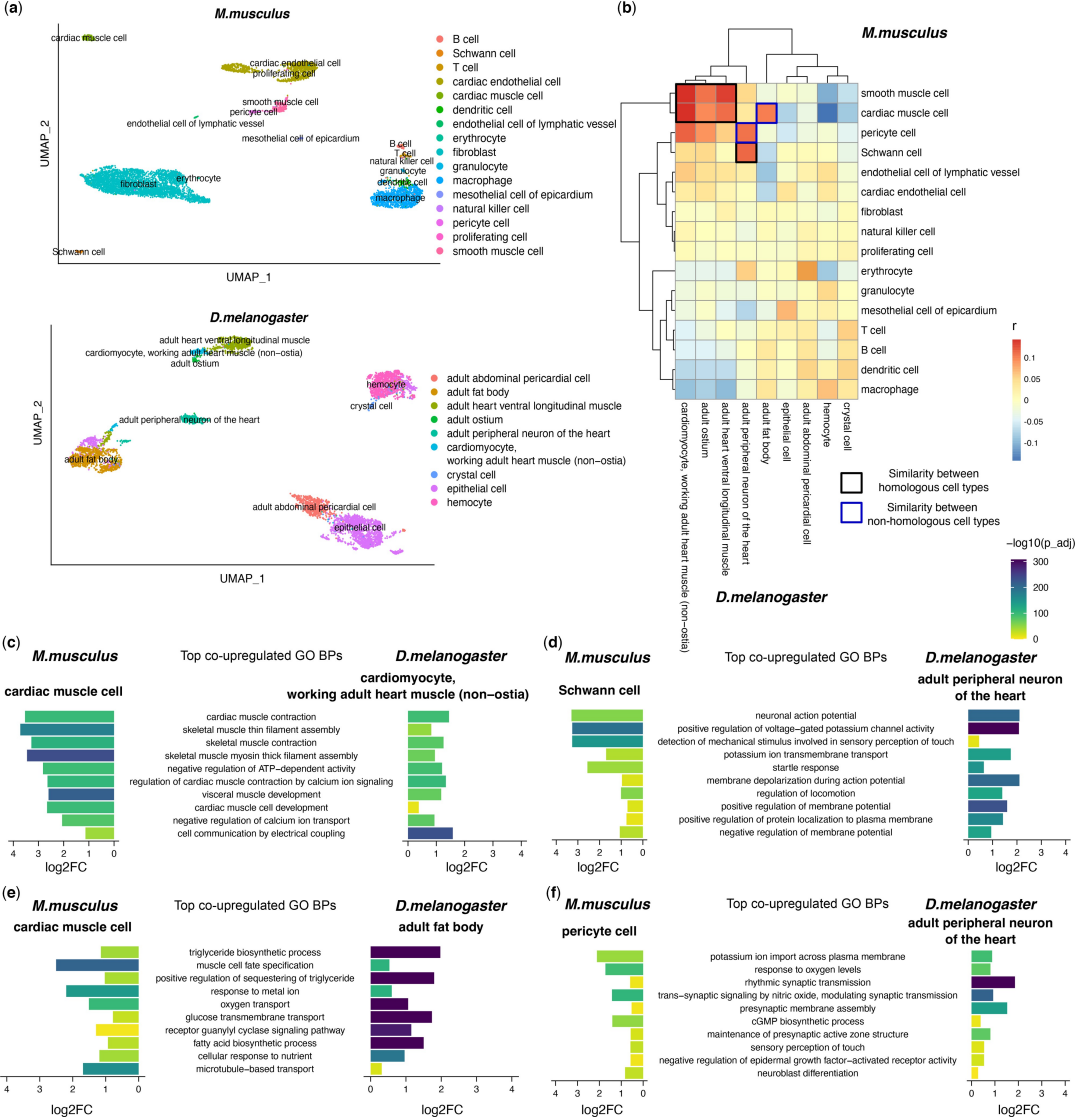
Comparing GO BP profile of cell types between mouse and fly heart. (a) UMAP visualization of the mouse (scRNA-seq) and fly heart (snRNA-seq) data under GO BP features. Annotated cell types from the original literature are shown. (b) Pearson’s correlation coefficient between mouse and fly heart cell types under GO BP profile. Outlines highlight muscle and neuron cell types, and similar but non-homologous cell types. (c–f) Shared top co-upregulated GO BP terms between mouse cell types (left) with fly cell types (right). The top 10 minimal GO BP terms ranked by average log2FC among the two species are shown. GO: gene ontology; BP: biological process; *r*, Pearson’s correlation coefficient; log2FC: log2 transformed fold change; −log10(p_adj): −log10 transformed adjusted *P*-value by Bonferroni correction; UMAP: UMAP: Uniform Manifold Approximation and Projection.

We set out to demonstrate scGOclust’s utility by analysing and comparing the heart tissue from mouse and fly (see Table 1 and Section 2 for dataset descriptions). Using GO BP annotations (the standard set, same applies to all following analyses) of genes in ENSEMBL and applying scGOclust, we observed that cell types clustered with GO BP form highly concordant clusters with their transcriptomic identity (Fig. 2a).

**Table 1.** Summary of datasets analysed in this study.

	Heart		Gut		Kidney		Whole-body	
	*M.musculus*	*D.melanogaster*	*M.musculus*	*D.melanogaster*	*M.musculus*	*D.melanogaster*	*C. elegans*	*H. vulgaris*
Number of analysed cells	7402	5086	19 962	9641	13 195	5731	154 251	24 458
Number of cell types	16	9	27	18	32	11	18	26
Number of analysed genes	21 127	12 674	14 688	13 407	19 125	11 353	7076	6792
Number of total GO BP features	12 304	5283	12 816	5642	12 284	5090	13 264	12 955
Number of shared GO BP features	4113		4502		4004		11 983	

Showing the number of cells and genes analysed in this study. Only included genes and cells that passed the original quality control from the source studies of the data. GO: gene ontology; BP: biological process.

Correlation analysis between mouse and fly heart cell types highlighted a high degree of similarity among muscle cell types and neuronal cell types, and a relatively weaker yet elevated similarity across blood cell types (Fig. 2b). Due to their excitatory nature, muscle and neuron cell types are also generally more similar. Subtracting the shared top co-upregulated GO BP terms between mouse and fly cardiac muscle cells revealed increased activity in cardiac muscle contraction, skeletal muscle filament assembly, and muscle development (Fig. 2c). For mouse Schwann cells and fly peripheral neurons of the heart, they share terms such as action potential, membrane depolarization, and ion transport-related, demonstrating their functional correspondence as neurons (Fig. 2d).

Interestingly, we also detected similarities between mouse cardiac muscle cells with fly fat body, as well as mouse pericyte cells and fly peripheral neurons (Fig. 2a). These two examples show shared key functional aspects between non-homologous cell types. Inspection of shared co-upregulated GO BP revealed that the former pair had increased fatty acid and glucose metabolic processes (Fig. 2e). This is in line with the fact that both cardiac muscles and fat tissues actively process fatty acids and glucose, either to mobilize or to store as the main source of energy. For the latter pair, top co-upregulated terms involve potassium ion import across the plasma membrane, response to oxygen levels and trans-synaptic signalling by nitric oxide (NO) (Fig. 2f). These processes reflect the key functional similarity of pericyte cells with neurons, as pericytes are excitatory cells whose active relaxation by NO/cGMP signalling modulates capillary dilation in response to blood oxygen level (Bergers and Song 2005, Attwell *et al.* 2016). These BPs have also been previously reported in fly peripheral sensory neurons (Broderick *et al.* 2003, Morton 2011, Rabinovich *et al.* 2016, Kozlov *et al.* 2020). However, currently, there is a lack of literature investigating NO signalling in the peripheral neurons of the fly heart.

### 3.2 Comparing GO BP standard set and stringent set

We compared the cross-species analysis results obtained using both the standard set and the stringent set of GO BP annotations. Overall, the observed patterns in the heatmap did not show significant changes (Supplementary Fig. S1), yet the relative strengths of mappings varied. There were 1221 and 1245 among 2000 highly variable features shared between the standard and stringent results, respectively, for mouse and fly, supporting that the cross-species results were similar. However, when considering the GO-by-gene binary matrix used in the analysis, we observed that 39% and 15% of annotations were dropped in the stringent set in cases of mouse and fly, respectively. This is in line with the portion of annotations excluded in the stringent set (Fig. 1). As a result, we conclude that relying solely on experimental evidence codes for the heart case yields comparable cross-species mapping results, but it also leads to a substantial reduction in the number of gene-to-GO BP annotations which might result in a less complete picture of the cell type functional profile.

### 3.3 Comparing scGOclust results with a conventional approach

As introduced, no other algorithms specifically leverage GO annotations to identify functionally corresponding cell types between species from scratch using scRNA-seq data. We show here that scGOclust provides more informative insights compared with a conventional *post hoc* analysis. Using the heart data as an example, we first performed GO BP enrichment using cell type marker genes for each species and calculated the percentage of intersecting terms among all enriched terms for each cell type pair. The harmonic mean of these percentages from the two species is shown in Supplementary Fig. S9. As observed, this conventional approach yielded only weak signals, such as between mouse enteroblasts and fly enterocytes. Furthermore, such an approach is prone to errors, as it does not account for contextual, cell type-specific biological roles. The apparent similarity between mouse intestinal enterocytes and the fly fat body was driven solely by the shared upregulation of basic metabolic pathways, which are not specific to cell type functions. Consequently, the conventional strategy was not informative in these cases.

### 3.4 Mapping cell types between the fly gut and mouse stomach and intestine

We proceeded to analyse and compare the GO BP profile of cell types between the mouse stomach and intestine (hereafter referred to as ‘mouse gut’) and fly gut. Unlike the heart, gut datasets mainly contained structural or secretory epithelial cell types, leading to overall more subtle cell type distinctions. It thus possessed more challenges for comparing cells between phylogenetically distant species from a functional perspective.

**Figure 3.**
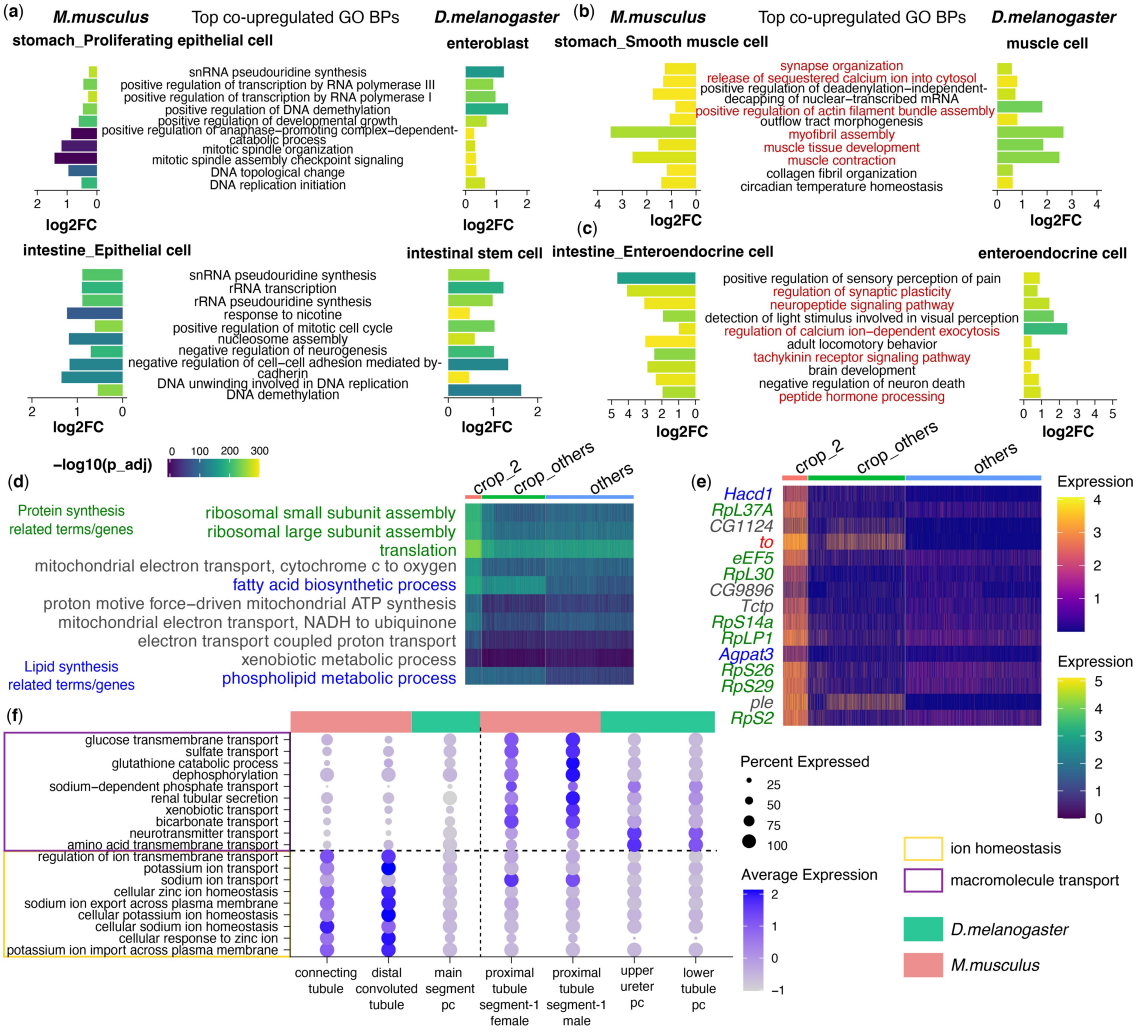
Comparing GO BP profile of cell types and subtypes between mouse and fly gut and kidney. (a–c) Shared top co-upregulated GO BP terms between mouse cell types (left) with fly cell types (right) in gut. The top 10 minimal GO BP terms ranked by average log2FC among the two species are shown. Highlighted terms in (b) and (c) are directly related to endocrine and muscle function. (d) Heatmap of top 10 up-regulated GO BP terms in crop_2, compared with other crop cells in the fly gut. (e) Heatmap of the expression level of top 15 up-regulated genes in crop_2, compared with other crop cells in the fly gut. In (d) and (e), protein synthesis or lipid synthesis-related BPs or genes are highlighted. The *to* gene highlighted in (e) is discussed in the results. (f) Comparison of co-upregulated terms between the mouse connecting tubule and distal convoluted tubule with the fly main segment PC, and between the mouse proximal tubule with the fly upper ureter and lower tubule PC in kidney. Boxes distinguish between genes involved in ion homeostasis, a shared function of the former group, and genes related to macromolecule transport, a shared function of the latter group. Expression values in (d)–(f) are scaled expressions. GO: gene ontology; BP: biological process; *r*, Pearson’s correlation coefficient; log2FC: log2 transformed fold change; *M. musculus: Mus musculus*; *D. melanogaster: Drosophila melanogaster*; −log10(p_adj): −log10 transformed adjusted *P*-value by Bonferroni correction.

In line with our hypothesis, applying scGOclust analysis on mouse gut showed that stomach epithelial cell types in mouse were overall highly similar (Supplementary Fig. S2a). In fly, enterocytes and their progenitor were similar to each other (Supplementary Fig. S2b). Between the two species, we observed the strongest correlation among stem cell types, followed by muscle cell types and weaker signals among endocrine cell types (Supplementary Fig. S2c). Such patterns are in line with previous studies demonstrating that stem cells show significantly higher similarities across distant species (Tarashansky *et al.* 2021). We further dissected specific BPs contributing to such similarities. Cellular proliferation and division-related terms such as nucleotide synthesis, DNA demethylation, DNA unwinding, and mitotic spindle formation are co-upregulated between mouse and fly proliferating cells, further confirming their shared active proliferation (Fig. 3a). For muscle cell types, terms such as myofibril assembly and muscle contraction indicate their functional correspondence (Fig. 3b). Co-upregulated terms between mouse stomach endocrine cell and fly enteroendocrine cell reflected their shared secretory activity and regulation of nervous responses (Fig. 3c).

### 3.5 Subpopulations in the fly crop uncovered by comparing with mouse stomach

The fly crop has been known to structurally correspond to the mammalian stomach: it is a highly expandable organ where food is temporarily stored before passing to the midgut (Apidianakis and Rahme 2011, Buchon *et al.* 2013). Reassuringly, PCA analysis on the GO BP profile of fly gut highlighted that the first PC separates crop cells from other gut cell types with terms related to contractile activity (Supplementary Fig. S3). However, the GO BP profile of the crop in its entirety did not show a strong correlation with stomach epithelial cells or muscle cell types (Supplementary Fig. S4). As the crop cells appeared as a large and heterogeneous population in scRNA-seq (Supplementary Fig. S2b), we proceeded to perform sub-clustering on the two species data for mouse stomach epithelial cells types and fly crop, to bring out subpopulations that are functionally more similar.

Interestingly, we detected a specific subpopulation of crop cells in the crop_2 cluster (denote as crop_2 hereafter) which strongly correlated with some mouse stomach epithelial cells (Supplementary Fig. S2c). Top shared GO BP terms between the two species, and for crop_2 cells compared with other cells, including several processes in protein translation, active energy metabolism and lipid synthesis (Fig. 3d). To validate our findings and establish stronger correlations with existing literature, we analysed the gene expression profiles of cells from the crop_2 GO BP cluster. We observed that compared to other crop cells, crop_2 has significantly elevated expression of ribosomal proteins such as *RpL37A*, *RpL30*, *RpS14A*, and *RpLP1*; translation elongation factor *eEF5*; as well as several endoplasmic reticulum enzymes involved in phospholipid synthesis, such as *Hacd1* and *Agpat3* (Fig. 3e). All these up-regulated genes confirm the elevation of protein and lipid synthesis process in crop_2 from GO BP analysis. Notably, the *to* gene which participates in the circadian regulation of feeding behaviour (So *et al.* 2000) is also strongly up-regulated in crop_2. The crop of *Drosophila melanogaster* contains various types of cells, such as gland cells and muscle cells, which could potentially secrete molecules such as enzymes or hormones. However, there currently exists very limited literature on crop secretion (Dimitriadis and Papamanoli 1992). We reason that by comparing crop subclusters cross-species with mouse stomach epithelial cells, which show strong secretion activity, scGOclust unveiled the crop population that performs a certain degree of secretion to participate in nutrient sensing and feeding regulation.

### 3.6 ScGOclust proposes analogies between fly renal system and mouse kidney

To further demonstrate the unique perspective of using GO BPs as features, we ran scGOclust on the fly renal system and the mouse kidney dataset, where the authors performed scRNA-seq mapping using SAMap. SAMap is the first algorithm that is not constrained by one-to-one orthologs, and currently, the only algorithm that achieves mapping of cell types between *Drosophila* and mouse with literature support.

ScGOclust generated a clear profile of cell types using GO BP as features (Supplementary Fig. S5) and identified mappings between the mouse and fly kidney cell types (Supplementary Fig. S6). In the original SAMap analysis by Xu *et al.* (2022), the authors discussed nine pairs of cell type mappings. By examining the strongest mapping mouse cell type for each fly cell type, scGOclust results agreed with seven of these pairs with one pair as ambiguous and one pair in disagreement. Supplementary Figure S7 provides a side-by-side comparison of scGOclust and SAMap results and explains the agreeing pairs. The pair for which scGOclust disagreed with SAMap is the mapping of fly main segment principal cells (PCs) (Supplementary Figs S6 and S7). Contrary to SAMap’s alignment to the proximal tubules segments 1–3 in mouse, scGOclust identified a closer similarity to the distal convoluted tubules and connecting tubules instead (Supplementary Fig. S6), which was supported by the shared up-regulated terms which were related to electrolytes homeostasis, such as regulation of ion membrane transport; potassium ion transport; sodium ion transport, etc (Fig. 3f). Conversely, the shared up-regulated terms between fly lower tubule PCs and upper ureter PCs with mouse proximal tubules are those involved in macromolecule reabsorption, for instance, glucose transmembrane transport, amino acid transmembrane transport, neurotransmitter transport, etc (Fig. 3f). Notably, scGOclust’s mapping aligns with the known physiological roles of these cell types supported by literature evidence. The fly main segment PC mainly generates primary urine via isosmotic secretion of ions into the malpighian tubule, while the lower tubule and ureter perform reabsorption (Wang *et al.* 2004, Rodan 2019). In mouse, the distal convoluted tubule and connecting tubule selectively secrete or absorb ions and water according to the electrolyte balance, while reabsorption is performed by the proximal tubule (Zhuo and Li 2013, McCormick and Ellison 2015). Therefore, at the level of BPs, scGOclust was able to correctly resolve the functionally meaningful correspondence between segments of Drosophila Malpighian tubules and mouse kidney.

To further confirm the mapping, we observed the expression pattern of several fly main segment PC marker genes and their orthologous genes in mouse that are relevant to ion and water homeostasis (Supplementary Fig. S8a). Inwardly rectifying potassium channel *Irk1* and *Irk3* has multiple mouse orthologs, among which *Kcnj1* and *Kcnj10* were highly expressed in distal straight tubule, distal convoluted tubule, connecting tubule and collecting duct (Supplementary Fig. S8b). Fly calcium channel *Trpm* has mouse ortholog *Trmp6* which was highly expressed in distal convoluted tubule and nephron connecting tubule (Supplementary Fig. S8c). The WNK-SPAK/OSR pathway, crucial for chloride and potassium homeostasis in fly PCs, parallels its significance in the mouse distal convoluted tubule (Goldsmith and Rodan 2023). Fly *Wnk* showed broad expression in PCs, while its mouse orthologs *Wnk1* and *Wnk4* show elevated expression in distal convoluted tubule and nephron connecting tubule (Supplementary Fig. S8d). Similarly, Fly fray demonstrated broad expression in PCs while mouse ortholog *Stk39* showed elevated expression in distal straight tubule, distal convoluted tubule, connecting tubule, and collecting duct (Supplementary Fig. S8e). We found that fly V-type proton ATPase subunit gene *Vha26* and *VhaSFD* have orthologs *Atp6v1e1* and *Atp6v1h* in mouse, which were highly expressed in several intercalated cell types in nephron connecting tubule and collecting duct (Supplementary Fig. S8f). Furthermore, the fly aquaporins *Drip* and *Prip*, highly expressed in main segment stellate cells, have several orthologs in mouse with distinct expression specificity to intercalated cell types or PCs in collecting duct (Supplementary Fig. S8g). In summary, these results further support the shared importance in electrolyte balance, acid–base balance, and osmotic pressure regulation between fly main segment and mouse distal convoluted tubules and connecting tubules detected by scGOclust.

While the high-level function of fly main segment PCs in generating primary urine, scGOclust identifies a divergence from mouse Bowman’s capsule (macula densa, Supplementary Fig. S6). The dissimilarity arises from distinct molecular mechanisms, as the bowman’s capsule filtration is passive and based on size and charge (Scott and Quaggin 2015), while main segment PCs perform active isosmotic secretion (Rodan 2019). Enrichment in ion transporters notably guides the mapping of main segment PCs to distal convoluted tubule and connecting tubule, reflecting their shared importance in electrolyte–water homeostasis. This example reflects that in complex tissues such as the kidney, scGOclust was able to discern nuanced distinctions in procedural versus molecular functional analogy.

### 3.7 Mapping hydra and roundworm whole-body scRNA-seq data using orthology-inferred GO annotations

Phylogenetically, mouse and fly diverged ∼700 MYA at the protostomes–deuterostomes split. To explore even more distant comparisons, we applied scGOclust to *H. vulgaris* and *C. elegans*, which diverged at the cnidarian–bilaterian split. Since curated GO annotations were unavailable for the specific genome versions used in scRNA-seq mapping, we leveraged eggNOG-mapper v2 to infer GO terms by sequence orthology, testing scGOclust’s effectiveness on less-curated genomes with inferred GO annotations. Encouragingly, cross-species correlation analysis revealed strong mappings between stem and germline cells, as well as between neurons, with top co-upregulated GO terms capturing key BPs. While epithelial cells mapped at a group level (Supplementary Fig. S10), several pairs were highlighted that are relevant to secretory activities. This demonstrates the unprecedented result that even for highly divergent species, utilizing orthology-inferred GO enables meaningful mappings via scGOclust.

## 4 Discussion and conclusion

We present scGOclust, a computational tool that characterizes cell type functional similarities between species. While the algorithm can also operate on single-species and closely related species, here, we applied it to evolutionarily remote species to demonstrate its unique perspective. Using heart, gut, and kidney data from mouse and fly, we show that scGOclust generates informative functional profiles of cell types and captures the functional correspondence between related cell types. We also characterized signals of functional convergence among some non-homologous cell type pairs. Furthermore, scGOclust can map germline cells, neurons, and epithelial cell types between *H. vulgaris* and *C. elegans*, highlighting its potential for even more distant species comparisons.

The scGOclust analysis highlighted cell type functions previously studied in flies but have not been investigated in depth at the cell type level. By comparing with mouse pericytes, we found evidence of NO signalling in fly neurons in the heart. Analysing subtypes of fly crop cells has revealed computational evidence of peptide and lipid secretion activity, which is an aspect of the crop’s function that is not fully understood. Coupled with DE analysis, we hypothesize that these crop cells respond to circadian control of feeding behaviour by secreting peptides and lipids. These hypotheses encourage further studies to uncover the secretory activity in the crop and the regulation of such secretion by circadian rhythm.

Comparing scGOclust with SAMap showed broad agreement. However, scGOclust’s hypothesis on the analogy of main segment PCs with distal convoluted tubule and collecting tubule aligned better with the literature. While scGOclust focuses on function via BP activities, SAMap highlights homologous genes and paralog substitution. Both approaches offer valuable insights, and their combined use can enhance cross-species cell type mapping.

Since scGOclust by default uses curated GO BP, its output is influenced by the information represented in the annotations. The availability of such records is not limiting, as GO BP resources cover over 5200 species—far more than those with publicly available scRNA-seq data. Additionally, we show that orthology-inferred GO annotations can be used for custom genomes. While concerns about annotation quality and area unevenness may arise, scGOclust is designed to extract insights from existing data. To address potential biases, we consciously select records to include and transparently evaluate annotation impact. We further allow users to choose annotations based on evidence codes. It is important to note that the stringent annotation set is not inherently superior but offers an alternative. For non-model species, selectively incorporating electronic annotations can enhance exploratory analyses.

## Supplementary Material

btaf195_Supplementary_Data

## Data Availability

ScGOclust is publicly available on CRAN https://cran.r-project.org/web/packages/scGOclust/index.html and development versions are available on GitHub github.com/Papatheodorou-Group/scGOclust/. The package version 0.1.3 was used in the analysis in this article. Jupyter notebooks to perform analysis in this study are available via github.com/Papatheodorou-Group/scGOclust_reproducibility.

## References

[btaf195-B1] Apidianakis Y , Rahme LG. *Drosophila melanogaster* as a model for human intestinal infection and pathology. Dis Model Mech 2011;4:21–30. 21183483 10.1242/dmm.003970 PMC3014343

[btaf195-B2] Ashburner M , Ball CA, Blake JA et al Gene ontology: tool for the unification of biology. The Gene Ontology Consortium. Nat Genet 2000;25:25–9. 10802651 10.1038/75556 PMC3037419

[btaf195-B3] Attwell D , Mishra A, Hall CN et al What is a pericyte? J Cereb Blood Flow Metab 2016;36:451–5. 26661200 10.1177/0271678X15610340 PMC4759679

[btaf195-B4] Bergers G , Song S. The role of pericytes in blood-vessel formation and maintenance. Neuro Oncol 2005;7:452–64. 16212810 10.1215/S1152851705000232 PMC1871727

[btaf195-B5] Broderick KE , MacPherson MR, Regulski M et al Interactions between epithelial nitric oxide signaling and phosphodiesterase activity in *Drosophila*. Am J Physiol Cell Physiol 2003;285:C1207–18. 12853288 10.1152/ajpcell.00123.2003

[btaf195-B6] Buchon N , Osman D, David FPA et al Morphological and molecular characterization of adult midgut compartmentalization in *Drosophila*. Cell Rep 2013;3:1725–38. 23643535 10.1016/j.celrep.2013.04.001

[btaf195-B7] Cantalapiedra CP , Hernández-Plaza A, Letunic I et al EggNOG-mapper v2: functional annotation, orthology assignments, and domain prediction at the metagenomic scale. Mol Biol Evol 2021;38:5825–9. 34597405 10.1093/molbev/msab293 PMC8662613

[btaf195-B8] Carbon S , Ireland A, Mungall CJ et al, Web Presence Working Group. AmiGO: online access to ontology and annotation data. Bioinformatics 2009;25:288–9. 19033274 10.1093/bioinformatics/btn615 PMC2639003

[btaf195-B9] Cunningham F , Allen JE, Allen J et al Ensembl 2022. Nucleic Acids Res 2022;50:D988–95. 34791404 10.1093/nar/gkab1049 PMC8728283

[btaf195-B10] Dimitriadis VK , Papamanoli E. Functional morphology of the crop of *Drosophila auraria*. Cytobios 1992;69:143–52. 1505206

[btaf195-B11] “Evolution at the cellular level”. Nat Ecol Evol 2023;7:1155–6. 37400516 10.1038/s41559-023-02133-6

[btaf195-B12] Gaudet P , Dessimoz C. Gene ontology: pitfalls, biases, and remedies. In: Dessimoz C, Škunca N (eds.), The Gene Ontology Handbook. New York, NY: Springer New York, 2017, pp. 189–205. 10.1007/978-1-4939-3743-1_14 27812944

[btaf195-B13] Gene Ontology Consortium, Aleksander SA, Balhoff J et al The gene ontology knowledgebase in 2023. Genetics 2023;224:iyad031. 10.1093/genetics/iyad031 PMC10158837 36866529

[btaf195-B14] Ghaddar A , Armingol E, Huynh C et al Whole-body gene expression atlas of an adult metazoan. Sci Adv 2023;9:eadg0506. 37352352 10.1126/sciadv.adg0506 PMC10289653

[btaf195-B15] GO Consortium. *Guide to GO evidence codes*. 2023. https://geneontology.org/docs/guide-goevidence-codes/ (12 October 2023, date last accessed).

[btaf195-B16] Goldsmith EJ , Rodan AR. Intracellular ion control of WNK signaling. Annu Rev Physiol 2023;85:383–406. 36228173 10.1146/annurev-physiol-031522-080651

[btaf195-B17] Gramates LS , Agapite J, Attrill H et al FlyBase: a guided tour of highlighted features. Genetics 2022;220:iyac035. 10.1093/genetics/iyac035 PMC8982030 35266522

[btaf195-B18] Greene D , Richardson S, Turro E. ontologyX: a suite of R packages for working with ontological data. Bioinformatics 2017;33:1104–6. 28062448 10.1093/bioinformatics/btw763 PMC5386138

[btaf195-B19] Han X , Wang R, Zhou Y et al Mapping the mouse cell atlas by Microwell-Seq. Cell 2018;173:1307. 29775597 10.1016/j.cell.2018.05.012

[btaf195-B20] Hao Y , Hao S, Andersen-Nissen E et al Integrated analysis of multimodal single-cell data. Cell 2021;184:3573–87.e29. 34062119 10.1016/j.cell.2021.04.048 PMC8238499

[btaf195-B21] Jung M , Dourado M, Maksymetz J et al Cross-species transcriptomic atlas of dorsal root ganglia reveals species-specific programs for sensory function. Nat Commun 2023;14:366. 36690629 10.1038/s41467-023-36014-0 PMC9870891

[btaf195-B22] Kozlov A , Koch R, Nagoshi E. Nitric oxide mediates neuro-glial interaction that shapes *Drosophila* circadian behavior. PLoS Genet 2020;16:e1008312. 32598344 10.1371/journal.pgen.1008312 PMC7367490

[btaf195-B23] Li H , Janssens J, De Waegeneer M et al Fly cell atlas: a single-nucleus transcriptomic atlas of the adult fruit fly. Science 2022;375:eabk2432. 35239393 10.1126/science.abk2432 PMC8944923

[btaf195-B24] Liu K , Deng S, Ye C et al Mapping single-cell-resolution cell phylogeny reveals cell population dynamics during organ development. Nat Methods 2021;18:1506–14. 34857936 10.1038/s41592-021-01325-x

[btaf195-B25] McCormick JA , Ellison DH. Distal convoluted tubule. Compr Physiol 2015;5:45–98. 25589264 10.1002/cphy.c140002 PMC5810970

[btaf195-B26] McLellan MA , Skelly DA, Dona MSI et al High-resolution transcriptomic profiling of the heart during chronic stress reveals cellular drivers of cardiac fibrosis and hypertrophy. Circulation 2020;142:1448–63. 32795101 10.1161/CIRCULATIONAHA.119.045115 PMC7547893

[btaf195-B27] Moreno P , Fexova S, George N et al Expression atlas update: gene and protein expression in multiple species. Nucleic Acids Res 2022;50:D129–40. 34850121 10.1093/nar/gkab1030 PMC8728300

[btaf195-B28] Morton DB. Behavioral responses to hypoxia and hyperoxia in *Drosophila* larvae: molecular and neuronal sensors. Fly 2011;5:119–25. 21150317 10.4161/fly.5.2.14284 PMC3127060

[btaf195-B29] Rabinovich D , Yaniv SP, Alyagor I et al Nitric oxide as a switching mechanism between axon degeneration and regrowth during developmental remodeling. Cell 2016;164:170–182. 26771490 10.1016/j.cell.2015.11.047 PMC5086089

[btaf195-B30] Ransick A , Lindström NO, Liu J et al Single-cell profiling reveals sex, lineage, and regional diversity in the mouse kidney. Dev Cell 2019;51:399–413.e7. 31689386 10.1016/j.devcel.2019.10.005 PMC6948019

[btaf195-B31] Rhee SY , Wood V, Dolinski K et al Use and misuse of the gene ontology annotations. Nat Rev Genet 2008;9:509–15. 18475267 10.1038/nrg2363

[btaf195-B32] Rodan AR. The Drosophila Malpighian tubule as a model for mammalian tubule function. Curr Opin Nephrol Hypertens 2019;28:455–64. 31268918 10.1097/MNH.0000000000000521 PMC6669090

[btaf195-B33] Rosen Y , Brbić M, Roohani Y et al Toward universal cell embeddings: integrating single-cell RNA-seq datasets across species with SATURN. Nat Methods 2024;21:1492–1500. 38366243 10.1038/s41592-024-02191-z PMC11310084

[btaf195-B34] Scott RP , Quaggin SE. Review series: the cell biology of renal filtration. J Cell Biol 2015;209:199–210. 25918223 10.1083/jcb.201410017 PMC4411276

[btaf195-B35] Sebé-Pedrós A , Saudemont B, Chomsky E et al Cnidarian cell type diversity and regulation revealed by whole-organism single-cell RNA-seq. Cell 2018;173:1520–34.e20. 29856957 10.1016/j.cell.2018.05.019

[btaf195-B36] Siebert S , Farrell JA, Cazet JF et al Stem cell differentiation trajectories in hydra resolved at single-cell resolution. Science 2019;365:eaav9314. 31346039 10.1126/science.aav9314 PMC7104783

[btaf195-B37] Smedley D , Haider S, Ballester B et al BioMart–biological queries made easy. BMC Genomics 2009;10:22. 19144180 10.1186/1471-2164-10-22 PMC2649164

[btaf195-B38] So WV , Sarov-Blat L, Kotarski CK et al takeout, a novel *Drosophila* gene under circadian clock transcriptional regulation. Mol Cell Biol 2000;20:6935–44. 10958689 10.1128/mcb.20.18.6935-6944.2000 PMC88769

[btaf195-B39] Song Y , Miao Z, Brazma A et al Benchmarking strategies for cross-species integration of single-cell RNA sequencing data. Nat Commun 2023;14:6495. 37838716 10.1038/s41467-023-41855-w PMC10576752

[btaf195-B40] Song Y , Papatheodorou I. scOntoMatch: *A*ligning *O*ntology *A*nnotation Across *S*ingle *C*ell *D*atasets with *‘*scOntoMatch*’. Version 0.1.1*. 2023.

[btaf195-B41] Tarashansky AJ , Musser JM, Khariton M et al Mapping single-cell atlases throughout Metazoa unravels cell type evolution. Elife 2021;10. 10.7554/eLife.66747 PMC8139856 33944782

[btaf195-B42] Wang J , Kean L, Yang J et al Function-informed transcriptome analysis of *Drosophila* renal tubule. Genome Biol 2004;5:R69. 15345053 10.1186/gb-2004-5-9-r69 PMC522876

[btaf195-B43] Wang R , Zhang P, Wang J et al Construction of a cross-species cell landscape at single-cell level. Nucleic Acids Res 2023;51:501–16. 35929025 10.1093/nar/gkac633 PMC9881150

[btaf195-B44] Wijesooriya K , Jadaan SA, Perera KL et al Urgent need for consistent standards in functional enrichment analysis. PLoS Comput Biol 2022;18:e1009935. 35263338 10.1371/journal.pcbi.1009935 PMC8936487

[btaf195-B45] Wu T , Hu E, Xu S et al clusterProfiler 4.0: a universal enrichment tool for interpreting omics data. Innovation (Camb) 2021;2:100141. 34557778 10.1016/j.xinn.2021.100141 PMC8454663

[btaf195-B46] Xu J , Liu Y, Li H et al Transcriptional and functional motifs defining renal function revealed by single-nucleus RNA sequencing. Proc Natl Acad Sci USA 2022; **119**:e2203179119. 10.1073/pnas.2203179119 PMC9231607 35696569

[btaf195-B47] Zhuo JL , Li XC. Proximal nephron. Compr Physiol 2013;3:1079–123. 23897681 10.1002/cphy.c110061 PMC3760239

