# Supplementary material for: ScGOclust: leveraging gene ontology to find functionally analogous cell types between distant species
Source: Bioinformatics. 2025 Jul 15;41(Suppl 1):i571–9. doi: 10.1093/bioinformatics/btaf195 (PMC12261464; doi:10.1093/bioinformatics/btaf195)
Supplement: btaf195_Supplementary_Data [file btaf195_supplementary_data.zip › btaf195_Supplementary_Data/BIOINF-2025-0921_alt_text.docx]

Alt text for: BIOINF-2025-0921 - ScGOclust: leveraging gene ontology to find functionally analogous cell types between distant species

Yuyao Song <ysong@ebi.ac.uk>

**Figure 1:**Graphical representation of the scGOclust method, showing cell type GO BP profile creation, normalisation, and cross-species comparison. A bar plot shows the distribution of GO BP evidence codes for mouse and fly, indicating those included in the standard and stringent sets.

**Figure 2:**Mouse and fly heart cell GO BP profile comparisons. Panel a shows a UMAP of both species using GO BP features. Panel b presents a cross-species correlation heatmap. Panels c–f highlight shared top co-upregulated GO terms for example mapping pairs between cell types, illustrating homology and convergence.

**Figure 3:**GO BP profile comparisons in mouse and fly gut and kidney. Panels a–c illustrate shared upregulated GO BP terms in gut cell types. Panels d–e show heatmaps of GO BP upregulation and gene expression in the crop_2 subcluster. Panel f compares shared kidney terms related to ion homeostasis and macromolecule transport.

**Table 1:**Overview of datasets used in this study, including the number of cells, cell types, genes, and GO BP terms included in the analysis.
