# Supplementary material for: ScGOclust: leveraging gene ontology to find functionally analogous cell types between distant species
Source: Bioinformatics. 2025 Jul 15;41(Suppl 1):i571–9. doi: 10.1093/bioinformatics/btaf195 (PMC12261464; doi:10.1093/bioinformatics/btaf195)
Supplement: btaf195_Supplementary_Data [file btaf195_supplementary_data.zip › btaf195_Supplementary_Data/Song.76.sup.1.pdf]

ScGOclust: leveraging gene ontology to find functionally analogous cell types between distant species

Yuyao Song[1,*], Yanhui Hu[2], Julian Dow[3], Norbert Perrimon[2,4], Irene Papatheodorou[1,5,6,*]

1 European Molecular Biology Laboratory-European Bioinformatics Institute (EMBL-EBI), Wellcome Genome Campus, CB10 1SD, Hinxton, UK

2 Department of Genetics, Harvard Medical School, 25 Shattuck Street, 02115, Boston, USA

3 School of Molecular Biosciences, University of Glasgow, G12 8QQ, Glasgow, UK

4 Howard Hughes Medical Institute, 4000 Jones Bridge Rd, Chevy Chase, 20815, Maryland, USA

5 Earlham Institute, Norwich Research Park, NR4 7UZ, Norwich, UK

6 University of East Anglia, Norwich Research Park, NR4 7UA, Norwich, UK

∗Corresponding author. ysong@ebi.ac.uk, irene.papatheodorou@earlham.ac.uk

Supplementary Material

Supplementary Figures 2

References 11

Supplementary Figures

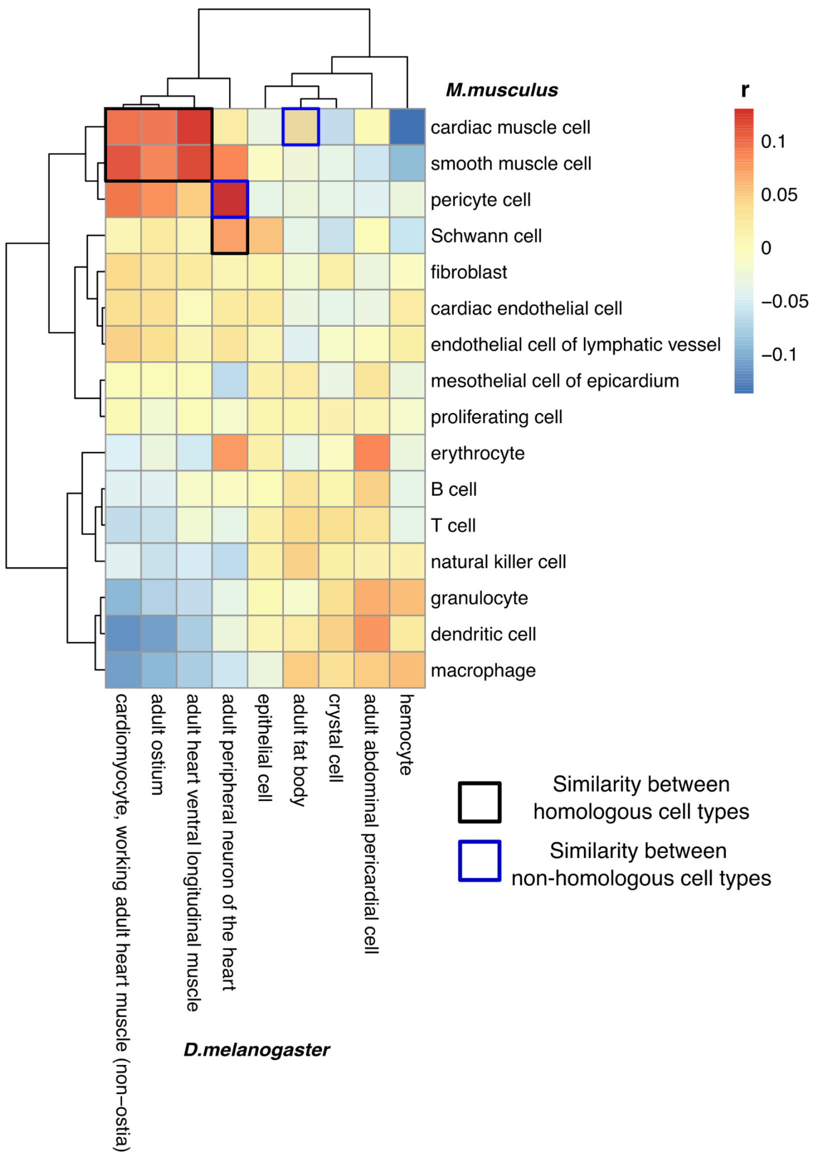

Supplementary Figure 1 Pearson's correlation coefficient between mouse and fly heart cell types under the stringent set of GO BP profile. Squares mark homologous or non-homologous cell type pairs discussed in the manuscript. r: Pearson's correlation coefficient.

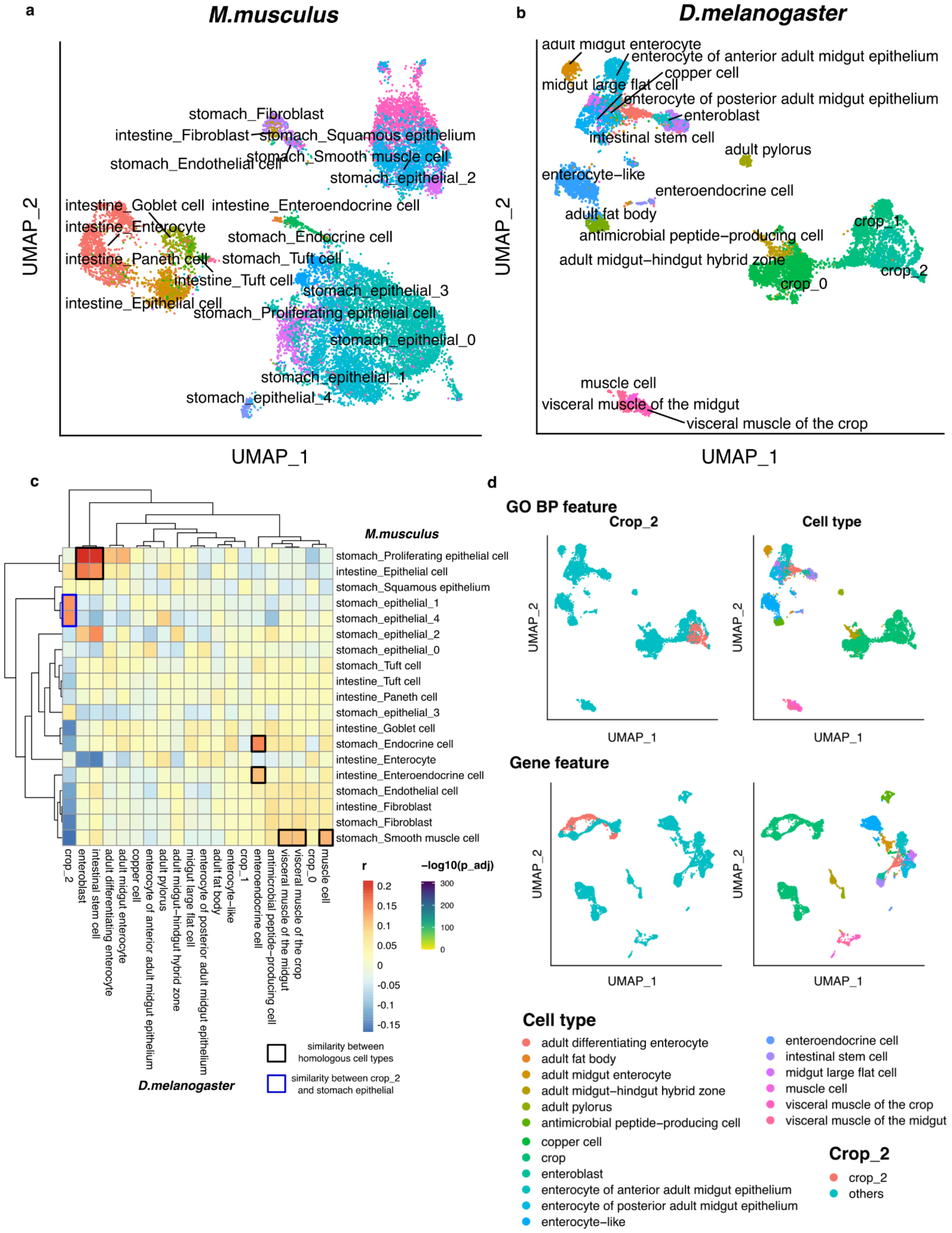

Supplementary Figure 2 Comparison between mouse gut and fly gut using scGOclust. (a)-(b), UMAP visualisation of the mouse stomach and intestine (Han et al., 2018), and the fly gut (Li et al., 2022) data under GO BP features. Annotated cell types from the original literature, as well as sub-clustering on mouse stomach epithelial cells and fly crop are shown. (c) Pearson's correlation coefficient between mouse and fly heart cell types under

GO BP profiles. The black box highlights similar cell types shown in d-f, and the blue box highlights the fly crop_2 cluster similar to some mouse stomach epithelial cells. (d) UMAP visualisation of the fly gut snRNA-seq data under GO BP features or gene features. Crop_2 corresponds to main Figure 3d, 3 GO: gene ontology; BP: biological process; r, Pearson's correlation coefficient; log2FC: log2 transformed fold change; *M.musculus: Mus musculus*; *D.melanogaster: Drosophila melanogaster;* -log10(p_adj): -log10 transformed adjusted p-value by Bonferroni correction; UMAP: UMAP: Uniform Manifold Approximation and Projection.

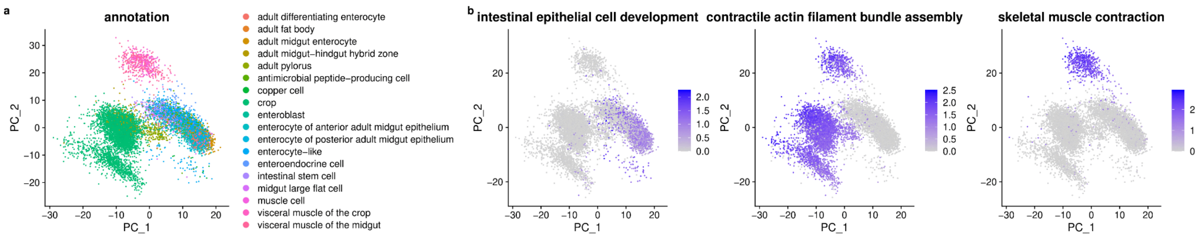

Supplementary Figure 3 PCA analysis of the fly gut data under GO BP features. (a) showing the first two PCs coloured by cell types and (b) showing the normalised activity of selected GO BP terms. PCA, principal component analysis; GO, gene ontology; BP, biological process.

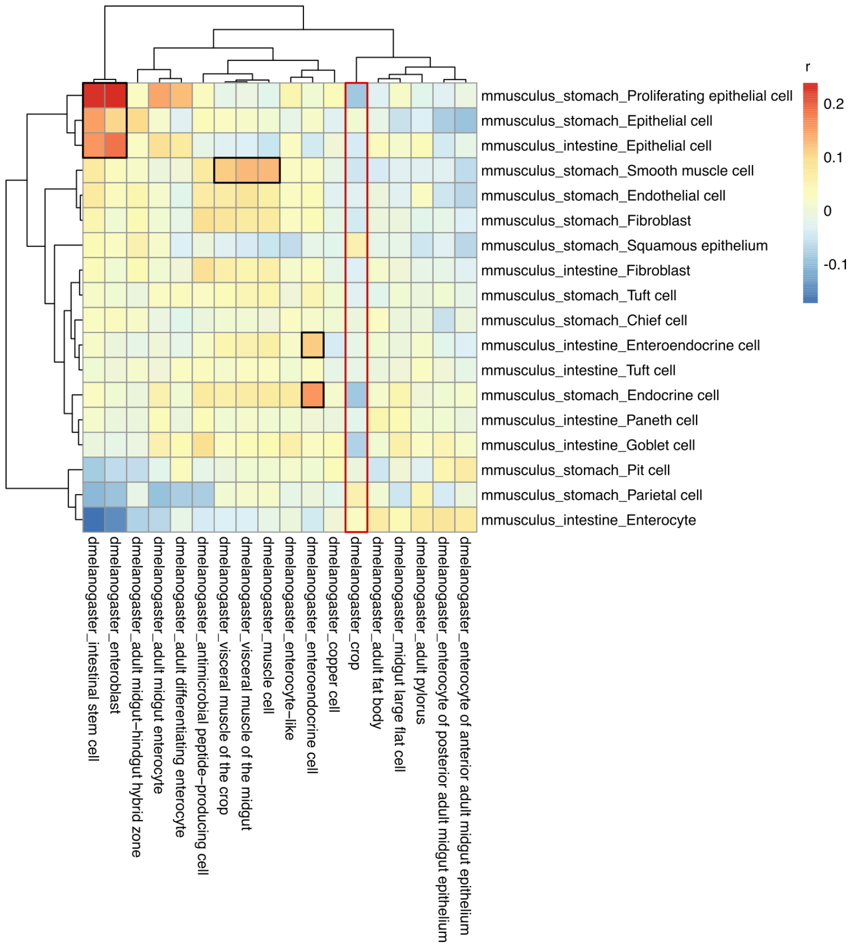

Supplementary Figure 4 Correlation of annotated gut cell types between mouse and fly. Heatmap showing Pearson's correlation coefficient between mouse and fly gut annotated cell types under GO BP profiles. The black box highlights related cell types, and the red box indicates crop cells in the fly. Crop cells in their entirety did not show a strong correlation with mouse gut cell types. *mmusculus: Mus musculus; dmelanogaster: Drosophila melanogaster*; r: Pearson's correlation coefficient.

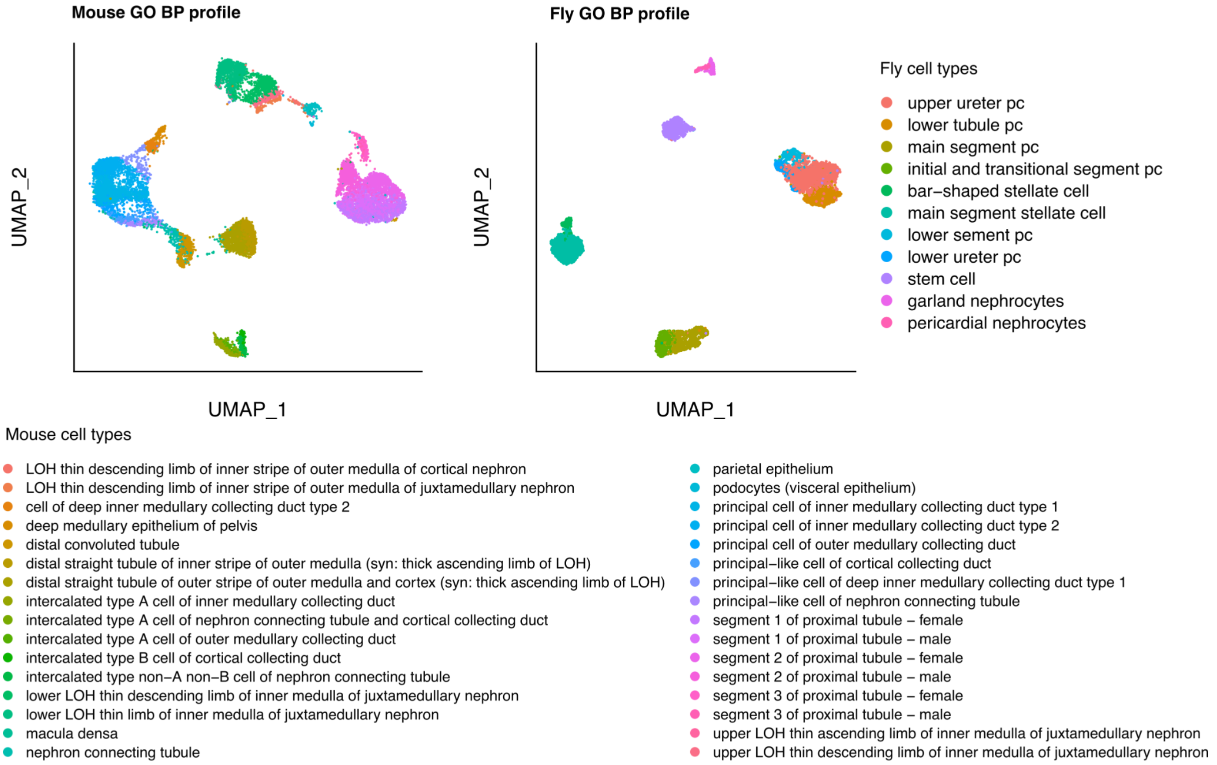

Supplementary Figure 5 ScGOclust profile of mouse kidney and fly renal system. UMAP visulisation showing that GO BP features effectively capture the cell type heterogeneity. GO: gene ontology; BP: biological process; UMAP: Uniform Manifold Approximation and Projection.

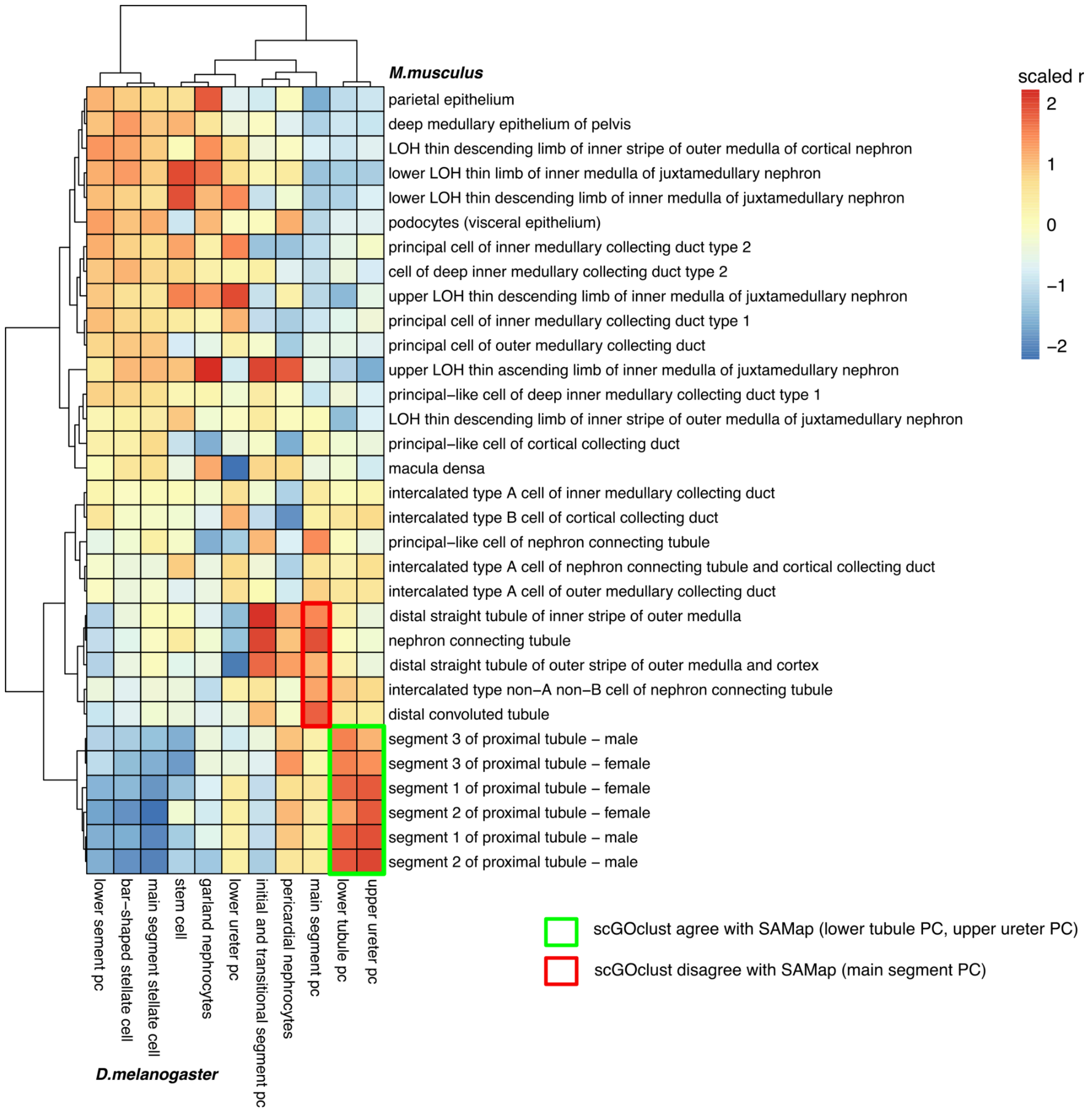

Supplementary Figure 6 Pearson's correlation coefficients scaled per column between mouse and fly kidney cell types under GO BP profiles. The green box highlights the lower tubule and upper ureter cell types that scGOclust agrees with SAMap, while the red box indicates the main segment P, which scGOclust maps to different cell types with SAMap. GO: gene ontology; BP: biological process; r: Pearson's correlation coefficient; *M.musculus: Mus musculus; D.melanogaster: Drosophila melanogaster*.

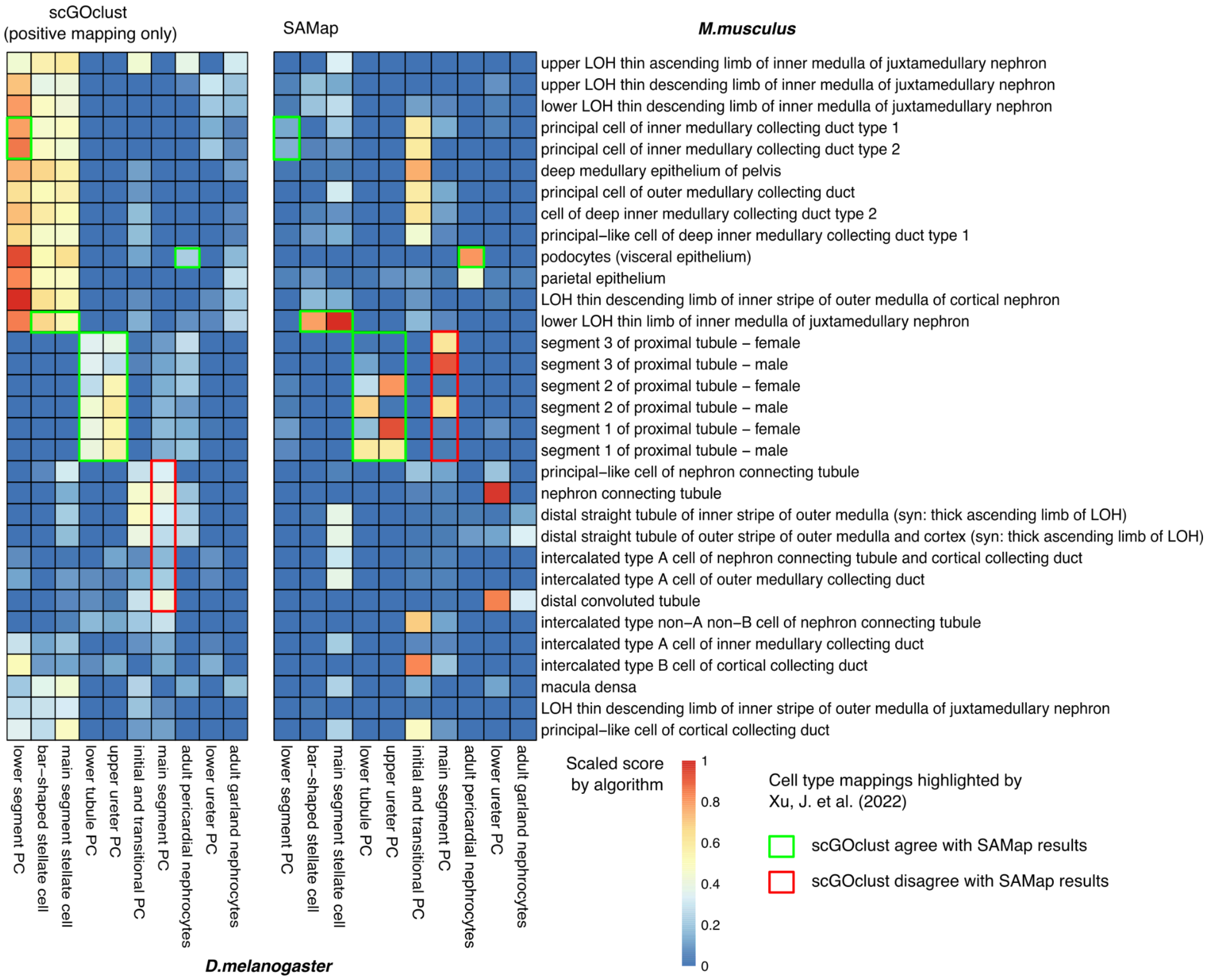

Supplementary Figure 7 Comparing scGOclust result with SAMap result for the mouse kidney and fly renal system dataset. A side-by-side comparison of scGOclust result and SAMap result on the same data (Xu et al., 2022). Since SAMap only calculates positive cross-species cell type mappings, only the positively correlated entries from scGOclust are included and shown. The scaled score by algorithm refers to the Pearson's correlation coefficient or the alignment score min-max scaled across the matrix for scGOclust (positive results only) or SAMap, respectively. We confirmed the mapping between fly lower tubule and upper ureter PC with mouse proximal tubules (segments 1 to 3); fly stellate cells with mouse lower LOH thin limb of inner medulla of juxtamedullary nephron, and fly lower segment PCs with mouse PCs of inner medullary collecting duct. Furthermore, scGOclust found the mapping between fly pericardial nephrocytes with mouse podocytes and fly garland nephrocytes with mouse parietal epithelium, but did not find a mapping between fly pericardial nephrocytes with mouse parietal epithelium. This further highlights the distinction between the two nephrocyte types, which is in line with the observation by SAMap. PC: principal cells; LOH: loop of Henle.

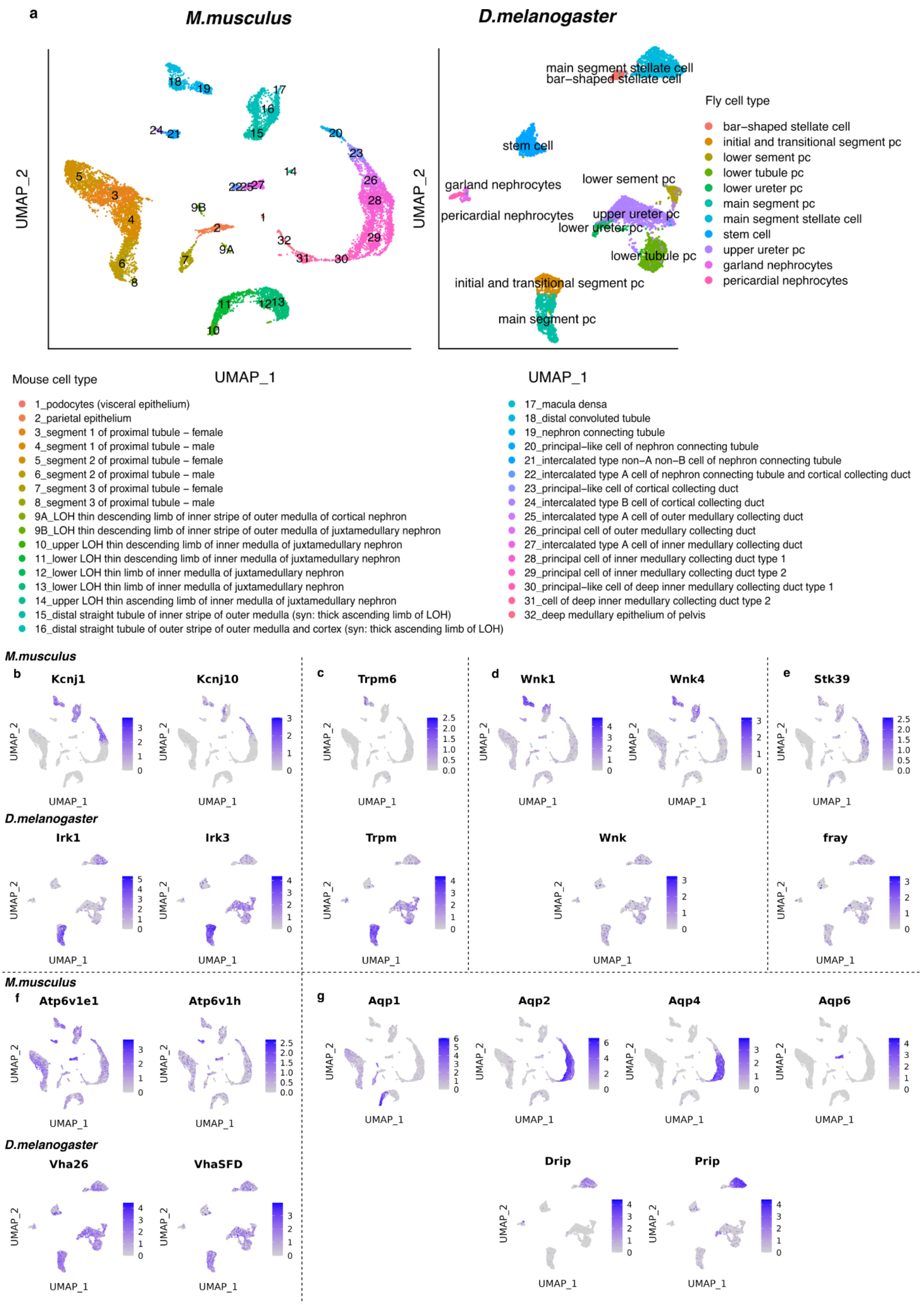

Supplementary Figure 8 Expression of orthologs in mouse and fly in the kidney.

Showing UMAP plots of the mouse and fly kidney dataset used in the study, coloured with

cell types (a) or scaled expression of selected genes involved in electrolyte and water homeostasis (b-g). UMAP: Uniform Manifold Approximation and Projection.

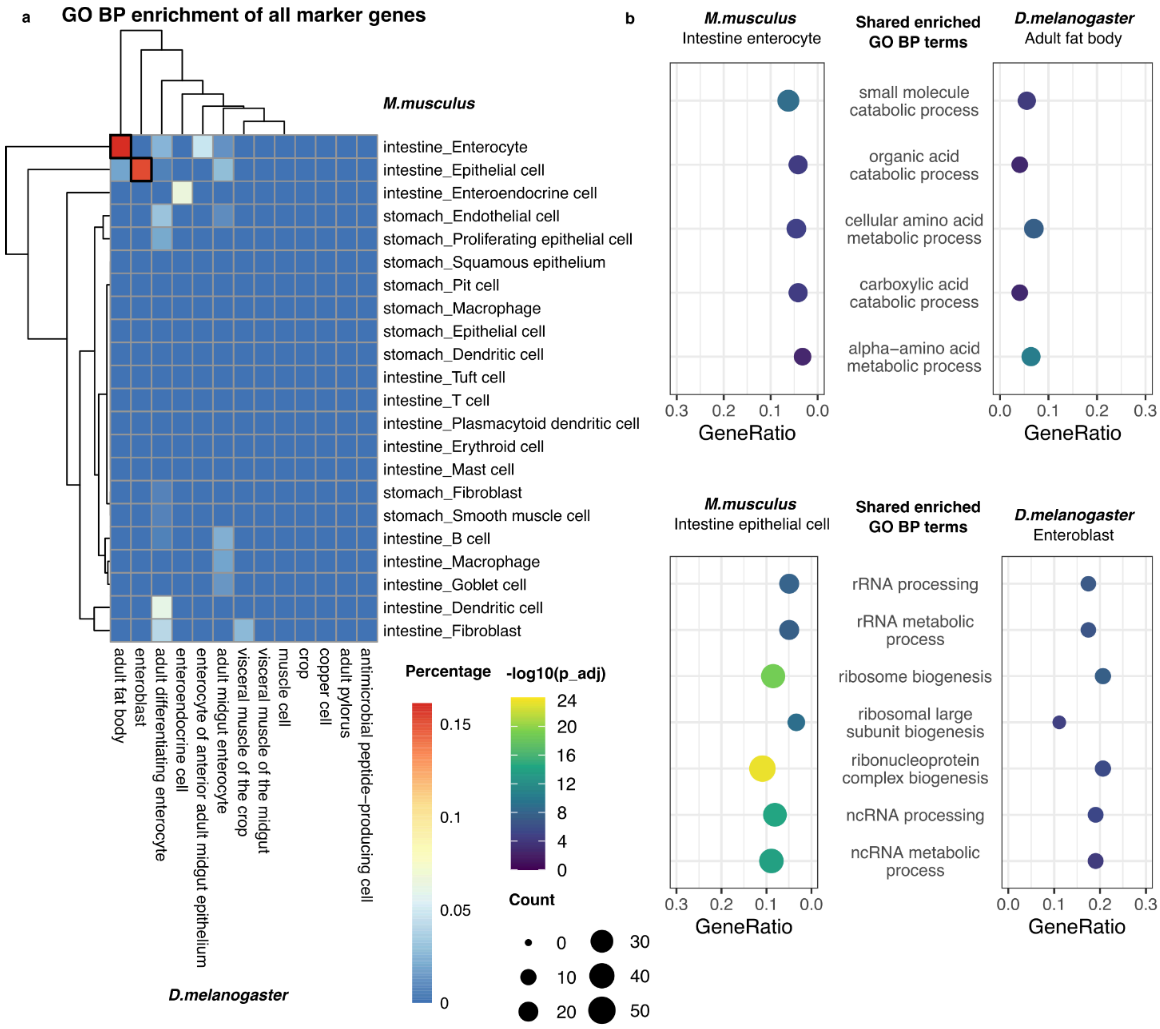

Supplementary Figure 9 Results from a conventional *post hoc* GO enrichment approach in mouse and fly gut. An example of a naive analysis to compare cell type GO BP enrichment across species, which gives uninformative results, while scGOclust analysis on the same data provided better results. (a) Heatmap showing the sharing of cell type-enriched GO BP terms between the mouse and fly gut. First, cell type marker genes were obtained from gene expression data, and GO BP enrichment was performed using these marker genes. We calculated the percentage of shared enriched terms for each pair of cell types and showed the harmonic mean between the two species. Black boxes highlight the positively correlated examples shown in (b). (b) Examples of the top shared terms in the GO BP enrichment result. -log10(p_adj): -log10 transformed adjusted p-value by Bonferroni correction; GO: gene ontology; BP: biological process.

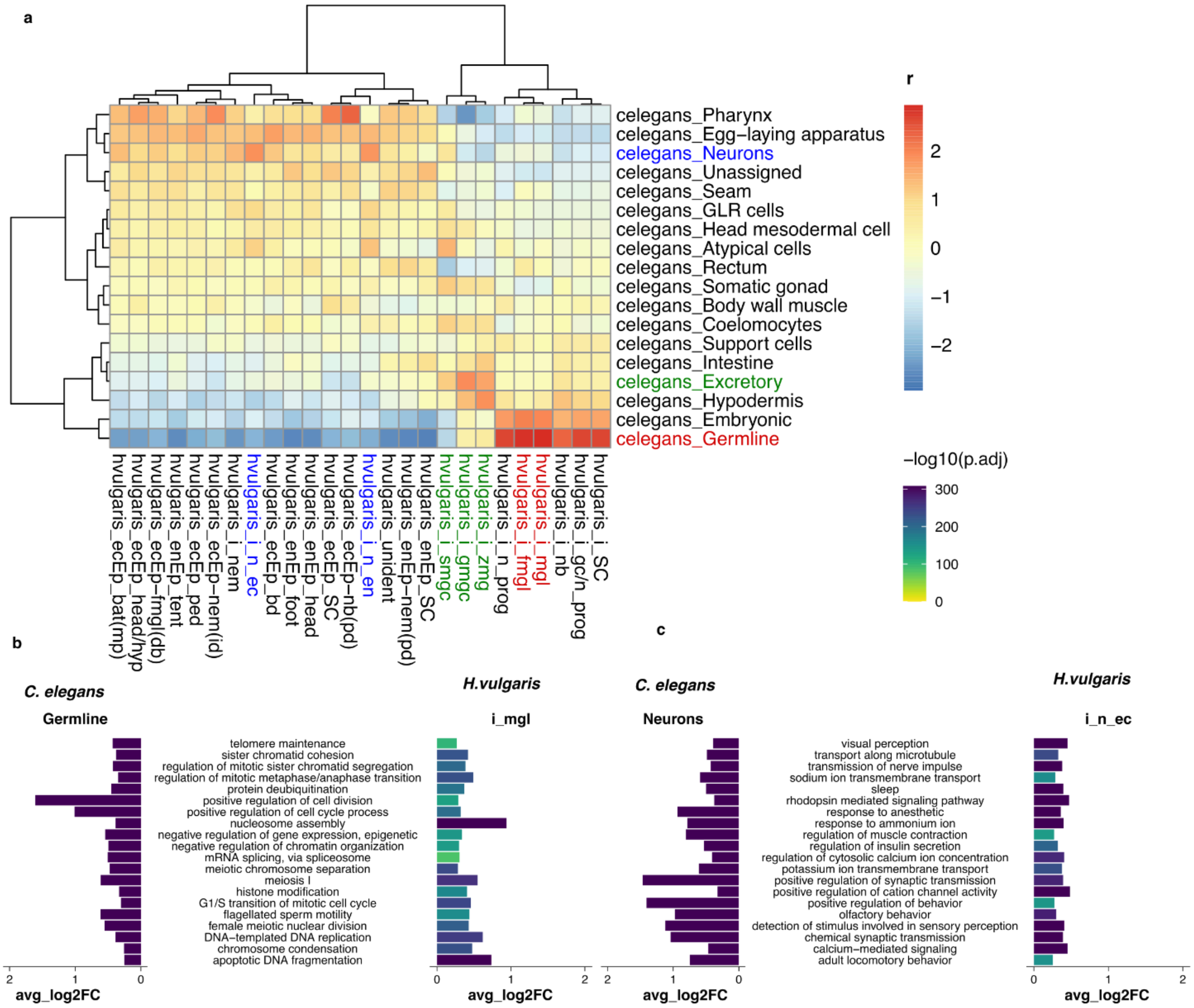

Supplementary Figure 10 Results from scGOclust analysis on whole-body scRNA-seq data from *C.elegans* and *H.vulgaris*. (a) correlation between the two species scaled per column, to find the most similar cell type in *C.elegans* respective to each *H.vulgaris* cell type. Colors highlight pairs including germline cells, neurons, and secretory cells. (b)-(c) Top co-upregulated terms between the two species cell types. Note that all terms in *C.elegans* have adjusted P value 0. C.elegans: *Caenorhabditis elegans*, H.vulgaris: *Hydra vulgaris*, -log10(p.adj): -log10 transformed adjusted p-value by Bonferroni correction, r: scaled Pearson's correlation coefficient, bat: battery cell, bd: basal disk, db: doublet cluster, ec: ectoderm, ecEP: ectodermal epithelial cell, en: endoderm, enEP: endodermal epithelial cell, fmgl: female germ-line, gc: gland cell, gmgc: granular mucous gland cell, i: cell of the interstitial lineage, id: integration doublet, mgl: male germline, mp: multiplet, nb: nematoblast, n: neuronal cell, nem: nematocyte, pd: suspected phagocytosis doublet, prog: progenitor, SC: stem cell, smgc: spumous mucous gland cell, tent: tentacle, zmg: zymogen gland cell.

References

Han, X., Wang, R., Zhou, Y., Fei, L., Sun, H., Lai, S., Saadatpour, A., Zhou, Z., Chen, H., Ye, F., Huang, D., Xu, Y., Huang, W., Jiang, M., Jiang, X., Mao, J., Chen, Y., Lu, C., Xie, J., … Guo, G. (2018). Mapping the Mouse Cell Atlas by Microwell-Seq. *Cell*, *173*(5), 1307.

Li, H., Janssens, J., Waegeneer, M. D., Kolluru, S. S., Davie, K., Gardeux, V., Saelens, W., David, F. P. A., Brbić, M., Spanier, K., Leskovec, J., McLaughlin, C. N., Xie, Q., Jones, R. C., Brueckner, K., Shim, J., Tattikota, S. G., Schnorrer, F., Rust, K., … Zinzen, R. P. (2022). Fly Cell Atlas: A single-nucleus transcriptomic atlas of the adult fruit fly. *Science*, *375*(6584), eabk2432.

Xu, J., Liu, Y., Li, H., Tarashansky, A. J., Kalicki, C. H., Hung, R.-J., Hu, Y., Comjean, A., Kolluru, S. S., Wang, B., Quake, S. R., Luo, L., McMahon, A. P., Dow, J. A. T., & Perrimon, N. (2022). Transcriptional and functional motifs defining renal function revealed by single-nucleus RNA sequencing. *Proceedings of the National Academy of Sciences*, *119*(25), e2203179119.
